# Supplementary material for: Simplified internal models in human control of complex objects
Source: PLoS Comput Biol. 2024 Nov 18;20(11):e1012599. doi: 10.1371/journal.pcbi.1012599 (PMC11723638; doi:10.1371/journal.pcbi.1012599)
Supplement: S1 Text — (DOCX) [file pcbi.1012599.s001.docx]

**S1 Text**

*I. Internal model of the coupled system: Multi-Mode*

This derivation starts with equations (6)-(8), which describe the linearized equations of motion of the cup-and-ball system coupled to the hand. Substituting equations (7)-(8) into equation (6) yielded:

$\left( m_{c}+m_{p} \right)\ddot{x}={-m}_{p}l\left( -\frac{g}{l}\phi-\frac{\ddot{x}}{l} \right)+F_{ff}+B\left( \dot{x}_{0}-\dot{x} \right)+K\left( x_{0}-x \right)$ (A1)

Simplifying terms led to:

$m_{c}\ddot{x}=m_{p}g\phi+F_{ff}+B\left( \dot{x}_{0}-\dot{x} \right)+K\left( x_{0}-x \right)$ (A2)

These equations were simulated as the internal model controlled by shaped inputs $[x_{0},\dot{x}_{0}]$ to generate desired cup and ball trajectories. Hence, we replaced the output variables $[x,\dot{x},\ddot{x}]$ and $\phi$ with $[x_{des},\dot{x}_{des},\ddot{x}_{des}]$ and $\phi_{des}$, respectively:

$m_{c}\ddot{x}_{des}=m_{p}g\phi_{des}+F_{ff}+B\left( \dot{x}_{0}-\dot{x}_{des} \right)+K\left( x_{0}-x_{des} \right)$ (A3)

Rearranging to solve for the feedforward force resulted in:

${F_{ff}=m}_{c}\ddot{x}_{des}-m_{p}g\phi_{des}+B\left( \dot{x}_{des}-\dot{x}_{0} \right)+K\left( x_{des}-x_{0} \right)$ (A4)

This is the force that, if applied to a system identical to the internal model, would result in the desired trajectories of the cup and ball.

This can be shown by applying the feedforward force generated by the multi-mode internal model as the input to the same model, achieved by substituting equation (A4) into equation (A2):

$m_{c}\ddot{x}=m_{p}g\phi+\left[ m_{c}\ddot{x}_{des}-m_{p}g\phi_{des}+B\left( \dot{x}_{des}-\dot{x}_{0} \right)+K\left( x_{des}-x_{0} \right) \right]+B\left( \dot{x}_{0}-\dot{x} \right)+K\left( x_{0}-x \right)$ (A5)

By simplifying and rearranging the remaining terms we obtained:

$m_{c}\ddot{x}-m_{p}g\phi+B\dot{x}+Kx=m_{c}\ddot{x}_{des}-m_{p}g\phi_{des}+B\dot{x}_{des}+Kx_{des}$ (A6)

The trivial solution to this equation is $x=x_{des}, \dot{x}=\dot{x}_{des}, \ddot{x}=\ddot{x}_{des}, \phi=\phi_{des}$.

When referring to feedforward force in the text $F_{ff}$, for simplicity, we called the non-impedance terms alone the feedforward force, obtaining equation (12), and noted that the shaped position and velocity profiles of the cup were simultaneously used as inputs to the impedance terms. Together, these input terms are equivalent to equation (A4).

*II. Internal model of the coupled system: Slow Mode*

This internal model was a mass-spring-damper system with the equivalent vibrational properties of the full system’s slower normal mode. Since we determined the equivalent modal stiffness, damping, and mass from the mode’s frequency and damping ratio, we had two equations and three unknowns. It was therefore impossible to uniquely determine the stiffness, damping, and mass simultaneously, and one of them had to be chosen.

We chose to fix the mass because we knew the actual values of the masses being manipulated in the experiment, while the actual stiffness and damping of the hand were unknown. Note as well that the mass scaled the values of Eqns. (13)-(14), but did not affect the mode shape itself. While the choice of modal mass is arbitrary to an extent and therefore up for debate, we believe it is reasonable to use the larger mass of a fourth-order system for the slower mode.

We took equation (15) and noted that the outputs of simulating the equations were the desired trajectories of the cup:

$m_{c}\ddot{x}_{des}+\tilde{B}_{slow}\left( \dot{x}_{des}-\dot{x}_{0} \right)+\tilde{K}_{slow}\left( x_{des}-x_{0} \right)=F$ (A8)

As before, we referred to the non-impedance term alone as the feedforward force, obtaining equation (16), while the shaped position and velocity profiles of the cup were used as inputs to the spring and damper in the full coupled system.

Note that if these inputs were applied to a system identical to this internal model, the resultant cup trajectories would be $[x_{des},\dot{x}_{des},\ddot{x}_{des}]$. When these inputs were applied to a system that is different from the internal model, such as the full, nonlinear coupled model described by equations (9)-(11), the spring and damper provided error correction forces. However, with finite-valued stiffness and damping coefficients, these forces were not large enough to fully recover the desired cup trajectories.

*III. Internal model of the coupled system: Fast Mode*

This derivation follows very closely to that of the slow mode internal model. For completeness, the equations of motion of the equivalent mass-spring-damper were:

$m_{p}\ddot{x}_{des}+\tilde{B}_{fast}\left( \dot{x}_{des}-\dot{x}_{0} \right)+\tilde{K}_{fast}\left( x_{des}-x_{0} \right)=F$ (A9)

Here, we obtained equation (17) describing the feedforward force as well as the desired position and velocity of the cup as inputs to the spring and damper. Note that the desired cup kinematics $[x_{des},\dot{x}_{des}, \ddot{x}_{des}]$ were generated by integrating the equations of motion of the internal model using shaped inputs specific to that model’s properties. Consequently, the desired trajectories differed for each internal model that was used. Thus, the feedforward force described by equation (17) was not equivalent to simply substituting $m_{p}$ for $m_{c}$ in equation (16), as $\ddot{x}_{des}$ differed in each equation.

*IV. Simplified internal model: Rigid Body*

As with the fast and slow mode internal models, a mass-spring-damper system was simulated. This system used the combined mass of the cup and ball as well as the actual stiffness and damping values of the hand:

$(m_{c}+m_{p})\ddot{x}_{des}+B\left( \dot{x}_{des}-\dot{x}_{0} \right)+K\left( x_{des}-x_{0} \right)=F$ (A10)

As before, the feedforward force was the term associated with cart acceleration, given by equation (18), while the shaped position and velocity profiles of the cup were used as inputs to the spring and damper in the full coupled system.

*V. Simplified internal model: No Impedance*

This internal model was based on the linearized cup-and-ball system without coupling to the hand, as described by equations (4)-(5). Noting that integrating these equations would result in the desired kinematics of the cup and ball, and that the resultant force would be the feedforward force we sought, we substituted $\ddot{x}_{des}$, $\phi_{des}$, and $F_{ff}$ for $\ddot{x}$, $\phi$, and $F$, respectively. Substituting equation (5) into equation (4) resulted in:

$\left( m_{c}+m_{p} \right)\ddot{x}_{des}={-m}_{p}l\left( -\frac{g}{l}\phi_{des}-\frac{\ddot{x}_{des}}{l} \right)+F_{ff}$ (A11)

Simplifying and solving for $F_{ff}$ yielded equation (19). Note that because the desired cup and ball trajectories were generated from different internal models, equations (12) and (19) are not equivalent. As with the other internal models, the shaped position and velocity profiles of the cup were used as inputs to the spring and damper in the full coupled system.

*Pre-Trimming Simulation Duration*

Experimental trials were trimmed at non-zero initial and final cup velocities. Each raw simulated trial began from zero-velocity initial conditions. In order for simulated trials to attain similar durations as well as non-zero initial and final cup velocities as in the corresponding experimental trials, simulations were initially conducted with longer durations and subsequently trimmed. The start of simulated trials was trimmed to the same initial cup velocity threshold as experimental trials were: 0.02 m/s. The end of simulated trials was trimmed so that its total duration was equal to the duration of the experimental trial being fit. In the optimization, the initial duration value was equal to that of the corresponding trimmed experimental trial. The optimization was then allowed to select simulated trial duration values up to 1000 ms longer than those of the trimmed experimental trial. This range was selected to be large enough to prevent solutions from clustering at the boundaries.

*RMSE Weights*

To compare RMSE values obtained using different variable weights, final RMSE values were divided by the sum of the square roots of all weights. Variable weights were chosen through an empirical process. First, the optimization was conducted with equal weights placed on all variables to establish a baseline. Next, separate optimizations were run with one variable weighted more heavily than the others to determine which variables had a larger impact on the objective function value. It was found that the cup and ball position variables had a greater impact than the velocity variables, which in turn had a greater impact than the acceleration variables. This may be due to greater noise content of recorded data in progressively higher derivatives of position variables. Based on the results of these individual optimizations, a “progressive” weighting scheme was implemented that weighted cup and ball position more than cup and ball velocity, which in turn were weighted more than cup and ball acceleration.
