# Supplementary figures and images for: Simplified internal models in human control of complex objects
Source: PLoS Comput Biol. 2024 Nov 18;20(11):e1012599. doi: 10.1371/journal.pcbi.1012599 (PMC11723638; doi:10.1371/journal.pcbi.1012599)

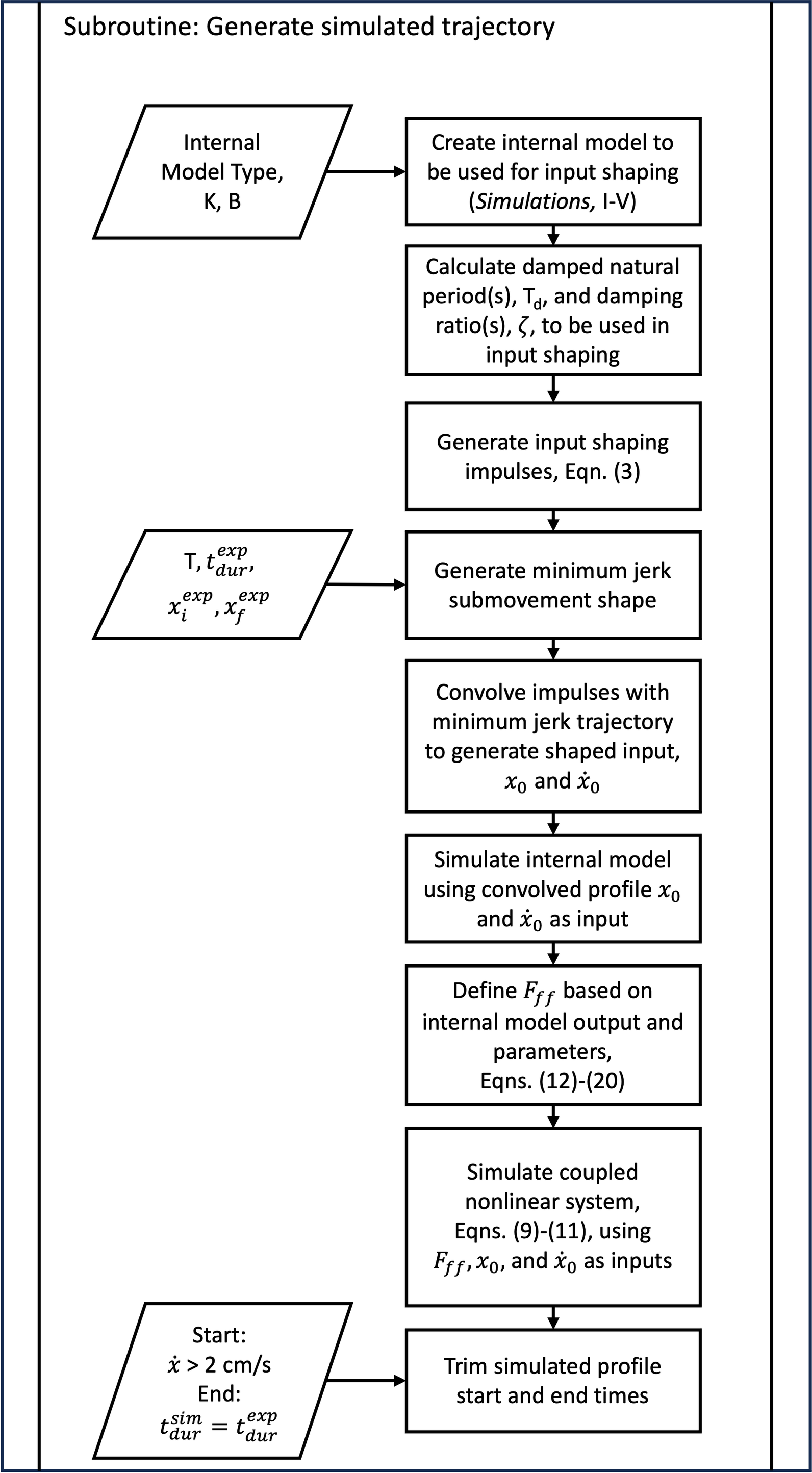

Supplement: S1 Fig — (TIF) [file pcbi.1012599.s002.tif]

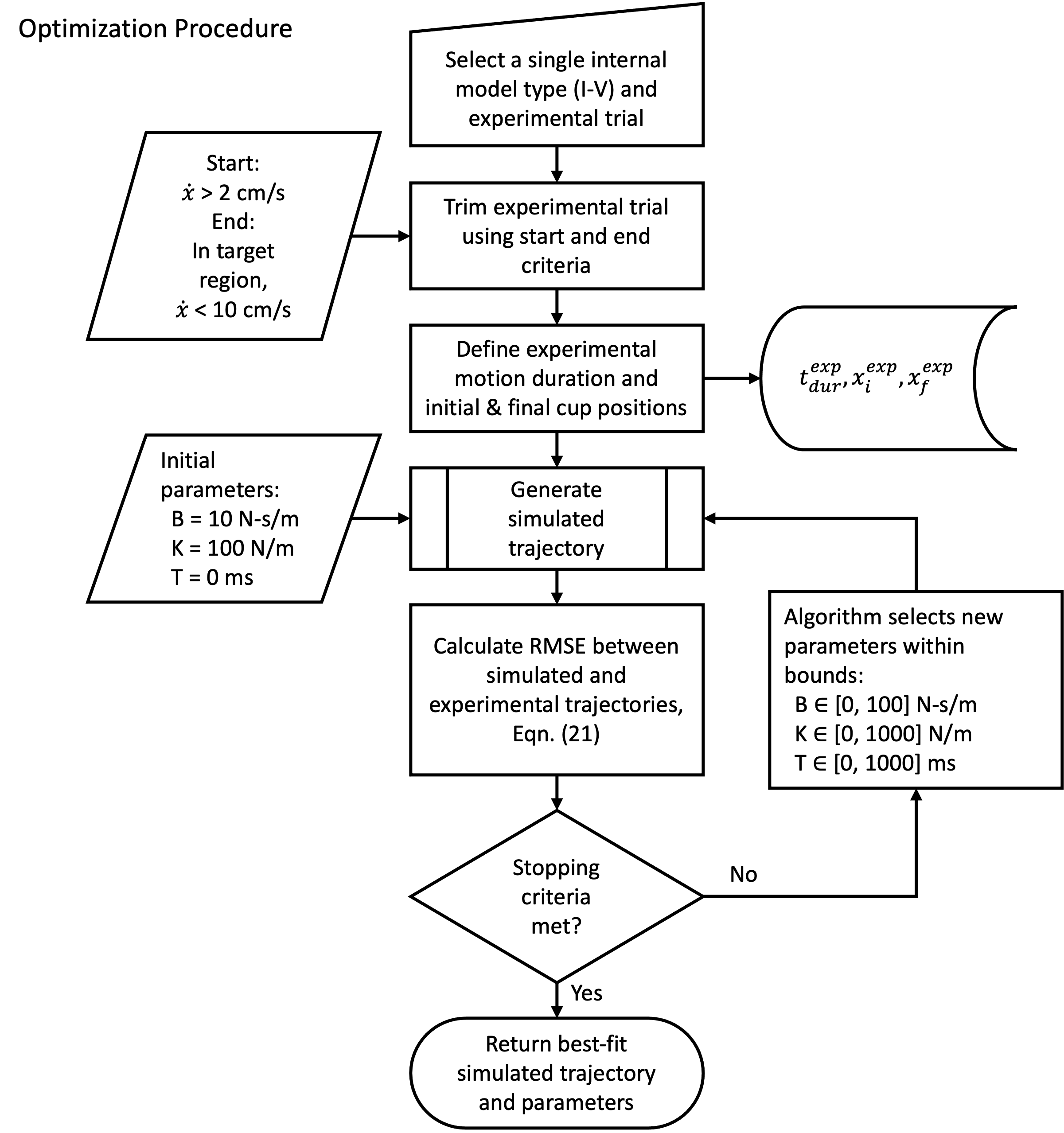

Supplement: S2 Fig — (TIF) [file pcbi.1012599.s003.tif]
