# Supplementary material for: Simplified internal models in human control of complex objects
Source: PLoS Comput Biol. 2024 Nov 18;20(11):e1012599. doi: 10.1371/journal.pcbi.1012599 (PMC11723638; doi:10.1371/journal.pcbi.1012599)
Supplement: Attachment — Submitted filename: Response to Reviewers.docx [file pcbi.1012599.s004.docx]

**Authors’ Response to Reviewers**

**Simplified Internal Models in Human Control of Complex Objects**

Salah Bazzi, Stephan Stansfield, Neville Hogan, and Dagmar Sternad

­We would like to thank the reviewers for showing interest in the work, commending the clarity and importance of the results, and the rigor of the approach. Their valuable feedback has helped us improve many aspects of the manuscript, as we detail below.

It is important to mention that upon revising the paper, we realized that some of the statistics used (specifically for the comparisons between the experimental data and the simulated models in Figs. 14, 15, and 16) were not appropriate. However, after redoing the statistical analyses the proper way, the results of the study were unchanged, namely that the Rigid Body internal model is the only model capable of reproducing the salient behavioral features observed in the data. In the course of this revised analysis, we also adapted some of the figures.

The reviewers’ comments are in black, and our responses below and edits to the manuscript are in red.

**Reviewer #1:**

At the very start, the motivation seems to talk primarily about tool use with novel objects but also touches on more familiar questions around internal models as in the control of one's own body. These are of course closely coupled but crucially different in how long one has to learn about a specific object/part and how stably that model can be reused. This impacts study design to follow in that there is currently a coupling between the learnability of a representation and its essential structure (if one had the privilege of learning over a long period of time as in the body)

We agree with the reviewer that learning an internal model of one’s limbs is a different problem than acquiring an internal model of a novel object. They occur at different timescales and potentially even involve different cognitive, sensory and motor processes. When interacting with a new object, learning/adaptation needs to be fast and on the fly. We have made this distinction clearer in the Discussion section (see page 29).

More specifically on the chosen task, i.e., the motivation of transporting a cup of coffee which is then reduced to a cup in ball and eventually a cart-pole control problem, it seems like the reduction is quickly losing many elements that make the initial motivation intriguing. Acknowledging up front that there is merit in studying control of the cart pole, what makes the cup of coffee task even more interesting to me is that the dynamics are not cleanly described by one or two fully observable variables, and the associated issues of internal degrees of freedom are really salient. This raises important questions about perception of the dynamics and its effects on representation learning. It would be helpful for the paper to better motivate this.

Note that the reduction of the problem was done for experimental tractability. Simulating a realistic 3-dimensional cup with sloshing coffee governed by nonlinear equations of fluid dynamics is computationally taxing. This computational complexity was undesirable as the study employed a virtual setup where real-time simulations of the system dynamics were required. Furthermore, using the cup-and-ball system, we were able to provide subjects with a performance metric (the residual ball angle) that was clearly defined and visually grounded. Using a sloshing liquid would have introduced an additional unnecessary complexity where subjects would have needed to learn a mapping between the liquid that they see, and the angle that they receive as feedback.

More importantly, even though this model simplifies the full dynamics of a cup of coffee, the essential challenges of the task were retained: underactuation and nonlinearity, where the latter could induce chaotic behavior.

We added the two points mentioned above to the Results section under *Experimental Task* (see page 6), and in the Methods section under *Experimental Apparatus and Task* (see page 34).

Moving on to the first main study with the cart pole. The authors begin with a baseline which is an input shaping controller based on a precise physical model. Almost surely, any internal representation of the dynamics would be imperfect with respect to this, but also it is unrealistic to expect anyone to have learnt this in a few trials. So, I find a crucial confounding between learning effects and essential structure here which could have been better disentangled, which would have improved the study.

We agree with the reviewer that one would not expect the humans to develop a precise physical model. However, supporting this claim with evidence is a necessary starting point, before delving deeper into the question of what is actually learnt. By analyzing the salient behavioral features in the deviations from this “perfect” model, we could start to postulate different structures of the internal model.

Furthermore, it is important to stress that subjects did not perform only “a few trials”. In fact, every subject interacted with the object for a total of 175 trials. The question of how the results would have differed if subjects were given even longer exposure to the object is a valid one and we discuss this in length in the Discussion section under *Simplified Models vs Uncertain Models*.

In terms of the essential structure, the authors have chosen a good framing in terms of input shaping. However, I wonder if the study would have also benefitted from a more qualitative description of the control task, which could inform some ways in which a human representation could be structured. For the cart-pole task, one description of the essential qualitative dynamics and global control of the nonlinear dynamics would be this sequence of papers:
- Åström, K. J., & Furuta, K. (2000). Swinging up a pendulum by energy control. Automatica, 36(2), 287-295.
- Kuipers, B., & Ramamoorthy, S. (2002, March). Qualitative modeling and heterogeneous control of global system behavior. In International Workshop on Hybrid Systems: Computation and Control (pp. 294-307). Berlin, Heidelberg: Springer Berlin Heidelberg.
- Ramamoorthy, S., & Kuipers, B. (2003, March). Qualitative heterogeneous control of higher order systems. In International Workshop on Hybrid Systems: Computation and Control (pp. 417-434). Berlin, Heidelberg: Springer Berlin Heidelberg

One advantage of this kind of qualitative constraint based description is that it gives more of a continuous parameterisation of control than the discrete categories in fig 13 and also suggest other ways in which a human cognitive representation would be structured without needing exact description of something like eq 4,5.

Looking forward in the paper, this might also provide an alternative parameterisation of the controller that respects essential qualitative constraints while explaining the lack of correlation mentioned in pp 22.

The choice of input shaping as the tool for addressing this question about internal models stems from the fact that input shaping is an exact solution to the experimental task of zeroing-out residual oscillations. With that consideration in mind, the control architectures put forth in the 3 papers suggested by the reviewer are not as relevant to the task at hand. Even though they act on the same system we consider in the paper, i.e., the cart-pendulum, these controllers seek to stabilize it at the unstable upright position, which is different from the task in our experiment where the pendulum mostly remains around its stable downright position.

Regarding the continuous and discrete parameterizations as they pertain to Fig.13, we would like to stress that the 5 internal models presented are a representative set, insofar as they included all structural simplifications of this system, which has only two dynamic modes of behavior. We arrived at them through a systematic reduction scheme, after careful study of the object’s dynamics. We now explicitly state this in the Simulations: Testing Different Internal Models section (see page 18).

In the same vein, the results around hyp 2 in pp 12 do not seem very surprising when one observes that almost surely the human representation is imprecise and also that the way they interpret the 'costs' might be quite different from the specific framing of the authors. So, I am not sure what to take from the numerical details such as peak ratios.

We agree with the reviewer that the fact that the human and simulated results are different is not very surprising in and of itself. However, the imprecision, while expected, is not of a random nature, but rather exhibits structure that can guide further investigation. There is a consistent and robust imprecision whereby the first peak is larger than the second peak. The peaks themselves are not interesting, but rather this asymmetry is one of the observable salient behavioral features that affords further insights into the structure of the internal model. We added a phrase to the manuscript to express this view (see last sentences under Hypothesis 2 on page 13).

All that said, the final observation about the need for damping and impedance control accords with my expectations in this domain, and is appropriately argued in the paper. However, I wonder if the experiment design could probe this more in terms of the ways in which feedforward term and damping are likely represented.

The system’s dynamics guided the experimental design. After careful study of the object and its properties, we designed the experiment and the task in a manner that would allow us to interrogate the structure of the internal model. Starting with one object with constant parameters in one experimental task, five different candidate internal models were identified, and the data analysis provided support for only one of the models.

In the future, one could certainly investigate other questions, such as how damping is represented as the reviewer suggests. However, we imagine that this would require devising an experiment where subjects interact with multiple objects with different damping properties, which is beyond the scope of this paper. Maintaining constant parameters was crucial in this study to facilitate differentiation between different structures of the internal model.

**Reviewer #2:**

The main question is how to generate the feedforward force F_ff. Four models are investigated, described by eqs. (12), (16), (18) and (19). These models seem to be inconsequent.

We assume the reviewer intended ‘inconsistent’. The five (not four) internal models are considered below.

First of all, a multiplier "g" (gravity) seems to be missing from eqs (1), (4), (6) and (9): m_p*g*l …
Also, a multiplier "l" (pendulum length) is missing from eqs (12) and (19): m_p*g*l …

We thank the reviewer for carefully inspecting the mathematical equations. We went back and rederived the equations of motion and found no mistakes. The system possesses two degrees of freedom and is therefore described by a set of two coupled differential equations. The multiplier “g” that is missing from eqs (1), (4), (6), and (9), is in fact present in the second equation of motion, namely (2), (5), (7), and (10).

For the missing “l” in eqs (12) and (19), we would like to note that the “l” cancels out when combining equations. To avoid confusion, we have now added the full derivation of the equations for the five different internal models in the Appendix (see pages 45-48).

The four models:

I. Multi-mode: eq. (12) is obtained from inverse dynamics. I guess, in eq. (12) the term
m_c*x"_des
should be
(m_c + mp)*x"_des
otherwise eq. (12) would be the same as (19).

The desired cup accelerations x’’_des differ in these two equations because they are each generated by forward-simulating the behavior of a different internal model. Thus x’’_des is different in each of the five equations given for F_ff. We have clarified this in the Appendix section (see pages 47-48) that explains the derivation of Eqn. (20) (Eqn. (20) was Eqn. (19) in the original submission; we added an earlier equation, increasing the numbering by one).

II. Slow mode
Here I think the cup is moved "together" with the ball, hence
F_ff = (m_c + mp)*x"_des
would be more appropriate than eq. (16).

Though the cup and ball move with the same frequency in the fast mode, they do not necessarily move “together” – there can be an amplitude and phase difference between the motions of different masses in a given normal mode. Since we are determining the equivalent modal stiffness, damping, and mass from the mode’s frequency and damping ratio, we have two equations and three unknowns. It is therefore impossible to uniquely determine the stiffness, damping, and mass simultaneously, and one of them must be chosen.

We chose to fix the mass because we knew the actual values of the masses being manipulated in the experiment, while the actual stiffness and damping of the hand were unknown. Note as well that the mass scales the values of Eqns. (13)-(14), but does not affect the mode shape itself. While the choice of modal mass is arbitrary to an extent and therefore up for debate, we believe it is reasonable to use the larger mass of a fourth-order system for the slower mode.

We added the above explanation regarding the choice of modal mass to the Appendix (see page 46).

III. Fast mode
Here I think the cup is moved but the ball remains in the same absolute position, hence
F_ff = m_c*x"_des
would be more appropriate in this case.

Our above response regarding the slow mode largely applies here as well. We would like to note, however, that both masses do move in the faster normal mode. The case in which one of the masses moves while the other remains still occurs at an anti-resonant frequency, which lies between the slow and fast mode resonant frequencies; we are instead deriving the slow and fast mode internal models from the resonance frequencies of the system. Again, the exact choice of modal mass is up for debate, but we believe it is reasonable to use the smaller mass for the faster mode.

IV. Simplified model
It is not clear whether F_ff is given by (18) or (19). Should rather be (18), which coincides with the Slow mode model. (19) can rather be considered as a simplified model of I. Multi-mode.

First, we must note that some of the confusion here may have inadvertently stemmed from the fact that the internal models were shown in Fig. 13 and ordered differently from the order that they were presented in the text. This ordering was corrected to be consistent in this revision.

In section IV, pertaining to the “Rigid Body” Simplified Internal Model, the equation for F_ff is given by Eqn. (19) (previously Eqn. (18)). Eqn. (20) (previously Eqn. 19) pertains to the “No Impedance” Simplified Internal Model. We hope that the expanded derivations of the internal model feedforward force equations added to the Appendix (pages 45-48) will resolve this misunderstanding.

Some other comments:
The ball is considered to be a material point (with mass but negligible dimension). Also, the underlying model consider a "mathematical pendulum" and not a physical one. These might be highlighted.

While this is true, we believe that the essential challenges inherent to controlling the object are not compromised, namely the underactuation and nonlinearity. Also, the visual representation and plausibility for the subject is not affected by this choice.

The parameters m_p and m_c strongly affects the difficulty of the task. How these values (1.1kg and 1.9kg) were chosen? Were the inertia of the manipulandum and that of the human arm considered?

Indeed, the choice of these parameters considered both the human and the robotic manipulandum. Regarding the subjects, the values were selected to be large enough such that subjects felt the forces generated by the ball, while also small enough that the subjects did not fatigue. With respect to the manipulandum, the only constraint was ensuring stability of the HapticMaster system. We now explicitly state the motivation behind these parameter choices in the Methods section under *Experimental Apparatus and Task* (see page 35).

The terms B and K in eq. (8) seem to be proportional and derivative control gains that are used in order to eliminate the error caused by the nonperfectly anticipated dynamics.

These mechanical impedance terms are equivalent to proportional and derivative control gains only under certain assumptions, e.g., perfect force-controlled actuation with zero-output mechanical impedance. However, that would be difficult to justify given known neuro-muscular behavior.

Some comments could be added about the overall closed-loop time delay (human response time + computer delay + possible additional delays). The overall delay in such a virtual task might range form 300ms to 600ms depending on the computer delay, so this might also affect the findings.

Over the past years we have conducted extensive experiments to measure the latency of the robotic interface and the visual projection. This time is computed as the time difference between the moment the robot’s handle moves until the time the movement is displayed on the screen. We have measured this to be 33 ms $\pm$ 17 ms, which is common to a range of experimental studies using a robotic interface and is assumed to have negligible impact on the results. We have now added this information in the Methods section under *Experimental Apparatus and Task* (see page 34 and 35).

The task itself and the results resembles the ball-and-beam balancing task, which is a same positioning task with cup radius being infinitely large hence resulting in a neutral open-loop system rather than a stable one:
<https://link.springer.com/article/10.1007/s00422-020-00815-z>

We thank the reviewer for pointing us to this paper. While the ball-and-beam system superficially resembles our cup-and-ball system, there are significant differences. First, our task involves translation of the cup, whereas the beam center is fixed in space. In addition, the ball-and-beam system is open-loop unstable (in the bounded-input, bounded-output sense) and the task is one of stabilizing the ball. In contrast, the cup-and-ball system is inherently stable; our task goal focuses on canceling residual oscillations. We have added this reference and other references on pendulum tasks to the Discussion section, and differentiated them from our study.

The overall results, that humans use simplified internal models is plausible, see, e.g., the simplified single-pendulum models for human standing still:
<https://doi.org/10.1371/journal.pone.0213870>

While interesting, we would like to point out that the “simplified single-pendulum” is mentioned in a different context and addresses a different problem than the one we investigate. That paper argues that the simplified pendulum is a good model of quiet standing, **not** that humans internally represent themselves as simplified pendulums when computing balancing torques and forces in quiet standing. Furthermore, the paper above is concerned with stabilizing the pendulum around an unstable state, which is not the case in our study.
